# Improvement of Selected Psychological Parameters and Quality of Life of Patients With Type 1 Diabetes Mellitus Undergoing Transition From Multiple Daily Injections and Self-Monitoring of Blood Glucose Directly to the MiniMed 780G Advanced Hybrid Closed-Loop System: Post hoc Analysis of a Randomized Control Study

**DOI:** 10.2196/43535

**Published:** 2023-01-24

**Authors:** Katarzyna Cyranka, Bartłomiej Matejko, Anna Juza, Beata Kieć-Wilk, Sabina Krzyżowska, Ohad Cohen, Julien Da Silva, Maxim Lushchyk, Maciej T Malecki, Tomasz Klupa

**Affiliations:** 1 Department of Metabolic Diseases Jagiellonian University Medical College Krakow Poland; 2 Department of Psychiatry Jagiellonian University Medical College Krakow Poland; 3 Hospital University in Krakow Krakow Poland; 4 Clinical Provincial Hospital of Frederic Chopin No 1 in Rzeszów Rzeszów Poland; 5 College of Medical Sciences University of Rzeszow Rzeszow Poland; 6 Medtronic Northridge California, CA United States; 7 Medtronic International Trading Sàrl Tolochenaz Switzerland; 8 Belarusian Medical Academy of Postgraduate Education Minsk Belarus

**Keywords:** type 1 diabetes mellitus, personal insulin pump, advanced hybrid closed loop, quality of life

## Abstract

**Background:**

While introducing new technologies and methods of treatment for type 1 diabetes mellitus (T1DM), it seems essential to monitor whether modern technologies in diabetes treatment may improve the psychological and emotional status of patients.

**Objective:**

This study aims to assess the baseline psychological parameters of patients with T1DM during investigation of the direct transition from multiple daily injections (MDI) and self-monitoring of blood glucose (SMBG) to the MiniMed 780G advanced hybrid closed-loop (AHCL) system and to evaluate changes in the psychological well-being and quality of life (QoL) after the transition in these individuals versus the control group.

**Methods:**

The trial was a 2-center, randomized controlled, parallel group study. In total, 41 patients with T1DM managed with MDI or SMBG were enrolled and randomized either to the AHCL or the MDI+SMBG group. Of these, 37 (90%) participants (mean age 40.3 years, SD 8.0 years; mean duration of diabetes 17.3, SD 12.1 years; mean hemoglobin A_1c_ [HbA_1c_] 7.2%, SD 1.0%) completed the study (AHCL: n=20, 54%; MDI+SMBG: n=17, 46%). Psychological parameters (level of stress, coping mechanisms, level of anxiety, self-efficacy level, acceptance of illness, locus of control of illness, life satisfaction, QoL) were measured at baseline and at the end of the study using 10 psychological questionnaires.

**Results:**

At baseline, the general level of stress of the examined patients was higher than in the general healthy Polish population (*P*=.001), but coping strategies used in stressful situations were significantly more effective and the level of self-efficacy (*P*<.001) was much higher than in the general population. The patients in this study accepted their illness more than patients with diabetes from the general Polish population (*P*<.001), but they felt that their health does not depend on them compared to the general population (*P*<.001). The overall life satisfaction was similar to that of the general population (*P*=.161). After 3 months from transition, the AHCL group reported an increase in 4 scales of the QoL—feeling well (*P*=.042), working (*P*=.012), eating as I would like (*P*=.011), and doing normal things (*P*=.034)—in comparison to the control group, where no significant change occurred. The level of both state anxiety and trait anxiety decreased in the AHCL group: State-Trait Anxiety Inventory (STAI) X1 scores (*P*=.009), STAI X1 stens (*P*=.013), and STAI X2 scores (*P*=.022). The AHCL group became more emotion oriented in stressful situations (Coping Inventory for Stressful Situations [CISS] E; *P*=.043) and significantly less self-blaming after 3 months of the study (*P*=.020).

**Conclusions:**

The results indicate that the patients who decided to take part in the transition study were characterized by higher levels of stress than the general healthy population but had better coping strategies and self-efficacy. Furthermore, transitioning from MDI+SMBG treatment to the AHCL in patients naive to technology may significantly improve psychological well-being and QoL within 3 months. The rapidity of these changes suggests that they may be related to the significant improvement in glycemic outcomes but also significantly less burdened diabetes self-management.

**Trial Registration:**

ClinicalTrials.gov NCT04616391; https://clinicaltrials.gov/ct2/show/NCT04616391

## Introduction

Diabetes care is a quickly evolving discipline, and numerous new technologies and recommendations have emerged [1]. Personal insulin pumps, continuous glucose-monitoring systems (CGMSs), and, most recently, hybrid closed-loop (HCL) systems, which are characterized by the coexistence of algorithm-driven automated insulin delivery combined with manual mealtime boluses, have had a major effect on the management of type 1 diabetes mellitus (T1DM) [2]. The popularity of these treatment options among patients with T1DM and recently also among patients with type 2 diabetes mellitus (T2DM) is increasing rapidly [3]. HCL systems are more advanced insulin delivery technologies that automatically increase, decrease, or suspend insulin delivery based on real-time continuous glucose-monitoring data [4-7].

One of this advanced hybrid closed-loop (AHCL) systems is the MiniMed 780G AHCL system (Medtronic), which has an algorithm that, in addition to automatically adjusting the basal insulin delivery, also delivers autocorrection boluses for high glucose levels [6-8]. Recent studies have evaluated the clinical effectiveness of the MiniMed 780G AHCL system in patients previously using advanced diabetes technologies, including continuous subcutaneous insulin infusion (CSII) or the CGMS or both combined to some degree in automated technologies [7,9-12]. To the best of our knowledge, no study so far has examined whether the MiniMed 780G AHCL system would be equally effective in patients who have no previous experience with the CSII/CGMS and how that shift would impact their psychological well-being.

Addressing the emotional aspects of diabetes and its management has received considerable attention in recent years. Diabetes distress can have a significant impact on the quality of life (QoL), disease management, and ability/willingness to respond to other diabetes interventions [13]. Studies suggest the importance of investigating the association between diabetes and negative emotional states and the psychological and psychopathological dimensions that may play a potential role in the therapeutic management of diabetes [14,15]. The American Diabetes Association’s (ADA) psychosocial position statement asserts that transitions throughout the lifespan and psychosocial barriers may impact disease management, and older adults with diabetes may experience unique age-related issues [16,17].

While introducing new technologies and methods of treatment for T1DM, it seems essential to monitor whether modern technologies in diabetes treatment may improve the psychological and emotional status of patients. This is an important factor also from the future perspective because these patients still have many years of life ahead [18]. To adequately assess the change in psychological parameters after the introduction of a new treatment method, we also considered it essential to check the preliminary psychological status of the patients in comparison to the general population.

The aim of this study was (1) to assess the psychological parameters of all the patients with T1DM included in this transition research in comparison to the general population and (2) to evaluate whether the transition from multiple daily injections (MDI) and self-monitoring of blood glucose (SMBG) directly to the MiniMed780G AHCL system has an impact on the psychological parameters of patients.

## Methods

### Studied Population

The study design is described in Ref. [7]. In summary, this 2-center, randomized controlled, parallel group study enrolled 41 patients with T1DM for at least 2 years. The patients were aged 26 to 60 years, with a hemoglobin A_1c_ (HbA_1c_) level below 10%, and treated with MDI and SMBG without any previous experience of CSII or CGMS technologies. Participants completed a 2-week run-in period, during which they demonstrated tolerance to wearing the sensor and compliance with a blinded CGMS and were randomly allocated to either the AHCL therapy (n=20, 49%) or continuation of MDI and SMBG therapy (n=21, 51%). Of these, 4 (10%) participants randomized to the MDI+SMBG group withdrew from the study: 3 (75%) immediately after randomization were not satisfied with group allocation, and 1 (25%) became pregnant during the study. The other 37 (90%) participants completed the 3-month study.

The first part of the analysis aimed and checking the psychological parameters of all the patients who enrolled in the study and comparing them with norms for the general population. We wanted to check whether the patients have any special characteristics that encouraged them to, on the one hand, not use modern technologies for many years and, on the other hand, decide to take part in the research project.

The second part of the analysis aimed at verifying whether after 3 months of the study, the AHCL group in which the MiniMed 780G AHCL insulin pump was introduced became different in terms of psychological parameters from the group that continued the previous method of treatment based on MDI and SMBG. We also checked whether the QoL with diabetes changed between the 2 groups.

Participants’ psychological parameters and QoL were assessed with the following set of questionnaires at baseline and at the end of the study:

- Coping Inventory for Stressful Situations (CISS): This consists of 48 statements about different behaviors typical for people in distress. Subjects have to determine on a 5-point scale the frequency of a given behavior in stressful, difficult situations. Scores are formatted on 3 scales (T, Task-Oriented Scale; E, Emotion-Oriented Scale; and A, Avoidance Scale) and 2 subscales (D, Distraction Subscale of the Avoidance Scale, and SD, Social Diversion Subscale of the Avoidance Scale) [19].
- Brief Coping Orientation to Problems Experienced Inventory (Brief-COPE): This is a tool for examining healthy and sick adults. It consists of 28 statements describing 14 strategies (2 statements in each strategy). Most often, the method is used to measure dispositional coping (ie, the assessment of typical ways of reacting and feeling in situations of severe stress) [20].
- State-Trait Anxiety Inventory (STAI): This measures anxiety understood as a transient and situationally determined state of the individual and anxiety understood as a relatively stable personality trait. The STAI consists of 2 subscales, one (X1) measuring state anxiety and the other (X2) measuring trait anxiety. The items of the subscales are printed on the reverse sides of the same test sheet. Each subscale consists of 20 items, which the subject answers by selecting 1 of 4 precategorized answers [21].
- Generalized Self-Efficacy Scale (GSES): This scale is a self-report measure of self-efficacy. The GSES is correlated to emotion, optimism, and work satisfaction. Negative coefficients are found for depression, stress, health complaints, burnout, and anxiety [22].
- Perceived Stress Scale 10 Items (PSS-10): This is widely used for measuring psychological distress [23].
- Satisfaction with Life Scale (SWLS): This is a measure of global life satisfaction. Scores on the SWLS correlate moderately to highly with other measures of subjective well-being and correlate predictably with specific personality characteristics [24].
- Acceptance of Illness Scale (AIS): The AIS can be used to measure illness acceptance in any condition. The scale consists of 8 statements describing the negative consequences of poor health, limitations imposed by disease, lack of independence, dependence on others, and lowered self-esteem [25].
- Multidimensional Health Locus of Control (MHLC) Scale Form C: The MHLC Scale Form C is an 18-item, general-purpose, condition-specific locus-of-control scale that can easily be adapted for use with any medical or health-related condition. It consists of 3 subscales: Internality, Doctors and Powerful Others, and Chance [26].
- Patient Requests Form (PRF): This is a list of patient expectations. Statements included in the PRF are composed of 3 factors concerning expectations connected with explanation of the disease, looking for support, and obtaining information about examinations and treatment [27].
- Quality of Life in Diabetes Questionnaire (QoL-Q Diabetes) [28]: This is 1 of the instruments to assess the QoL for adults with T1DM. The Polish version of the questionnaire was prepared based on the written content of the Mapi Research Trust, the copyright owner. Validation of the Polish version included forward translation by a health professional in clinical psychology and psychiatry, familiar with the terminology of the area covered by the instrument and with an MA in English philology; an expert panel analysis of items; back translation by a native speaker; and pretesting on a sample of patients with T1DM. The questionnaire is a self-assessment scale composed of 2 parts. The first measures the QoL with diabetes in a given (1 of 23) life area. In the second part, the patient assesses the importance of each of the 23 aspects of life on a 3D scale. The mean value of the global QoL is 138 points. The maximum test result is 345 points. The mean value for a given area is 6, while maximum for a given area is 15. The higher the result, the better the QoL assessed by the patient [7].

For comparisons with general Polish healthy population and the general Polish population of patients with diabetes, we used data collected by the Polish Test Laboratory of the Polish Psychology Association [19-27]. For each test, there was a representative group from the Polish population selected and examined by the psychological team. We chose data for healthy controls and patients with diabetes, stratified by age and sex, were appropriate, including the data provided by PTP statistical parameters (mean, SD, number of participants examined, etc), and we used proper statistical calculations for such analysis.

### Ethical Considerations

The study was approved by the bioethics committee (no. 1072.61201.8.2020, date May 28, 2020, trial registry no. NCT04616391). All patients provided written informed consent to participate in this study. The collected data were stored anonymously in an encrypted disc in the hospital according to bioethics committee recommendations. The participants did not receive any financial compensation for participation in the study.

### Statistical Analysis

To assess differences between the psychological scores for our group and the general population, a test for 2 means was used. To compare 2 independent variables, the Student or Welch *t* test for normally distributed (the Shapiro-Wilk test) continuous variables was performed, otherwise the Mann-Whitney *U* test was conducted. To compare 2 dependent groups, the paired *t* test or the Wilcoxon signed-rank test, when appropriate, was used. To test for the effect of treatment allocation on psychological outcomes, analysis of covariance (ANCOVA) was conducted, adjusting for treatment arm and baseline values. Once the significant interactions were confirmed, we included them into the model and then estimated the adjusted mean difference between the treatment arms (we centered the baseline value on the mean, and then the estimate in the arm was the adjusted mean difference between the treatment groups). When the ANCOVA assumption was not met, we performed the Wilcoxon rank-sum test. Analyses were performed with R version 4.1.0 and R Studio version 1.3.959 (R Core Team and the R Foundation for Statistical Computing).

## Results

### Participant Characteristics

Glycemic outcomes of the transition study have been presented previously [7] and include the time spent in target (time in range, TIR), which increased from mean 69.3% (SD 12.3%) at baseline to mean 85.0% (SD 6.3%) at 3 months in the AHCL group, while remaining unchanged in the control group: treatment effect 21.5% (95% CI 15.7%-27.3%), *P*<.001. The time below range (TBR; <70 mg/dL) decreased from mean 8.7% (SD 7.3%) to mean 2.1% (SD 1.7%) in the AHCL group and remained unchanged in the MDI+SMBG group: treatment effect mean –4.4% (95% CI –7.4% to –2.1%), *P*<.001 [7]. Baseline characteristics of the participants are presented in Table 1.

**Table 1:** Baseline characteristics of the participants (N=37) (adapted from Matejko et al [7] which is published under Creative Commons Attribution 4.0 International License [29]).

Category	MDI^a^+SMBG^b^ group (n=17, 46%)	AHCL^c^ group (n=20, 54%)	*P* value
Gender (female), n (%)	8 (47)	8 (40)	.920
Age (years), mean (SD)	40.9 (7.8)	39.8 (8.3)	.671
Diabetes duration (years), mean (SD)	17.6 (12.2)	17.1 (12.2)	.749
HbA_1c_^d^ level at enrollment (%), mean (SD)	7.4 (1.2)	7.05 (0.8)	.349
BMI (kg/m^2^), mean (SD)	25.6 (2.64)	24.5 (3.3)	.280
Body weight (kg), mean (SD)	77.7 (14.4)	76.3 (14.7)	.774

^a^MDI: multiple daily injection.

^b^SMBG: self-monitoring of blood glucose.

^c^AHCL: Advanced Hybrid Closed Loop.

^d^HbA_1c_: hemoglobin A_1c_.

### Results of the First Part of the Analysis

A comparison of the study participants with the general healthy population is presented here.

In the analysis, we observed that the T1DM group of 37 patients (study population), who volunteered to participate in our study, differed in many aspects of psychological parameters from the general healthy population.

Although the general level of stress in the study population measured with the PSS-10 was significantly higher in our study population than in the general population (mean 6.62, SD 7.50 vs mean 22.14, SD 4.27, *P*<.001), the coping strategies used by our study population turned out to be more effective in many aspects. In difficult and stressful situations, our study population is less likely to focus on emotions compared to the general population (mean 45.16, SD 9.94 vs mean 36.94, SD 10.65, *P*<.001) and does not avoid confrontation with a difficult situation (mean 19.11, SD 5.51 vs mean 16.34, SD 4.40, *P*=.002), as indicated by CISS results. Brief-COPE results revealed that the participants are much more active in coping with stressful events compared to the general population (mean 1.57, SD 0.79 vs mean 2.22, SD 0.69, *P*=.003) and are more effective in planning how to manage a stressful event (mean 1.89, SD 0.79 vs mean 2.30, SD 0.69, *P*=.005). Additionally, they more often use positive reframing compared to the general population (mean 1.67, SD 0.77 vs mean 1.89, SD 0.54, *P*=.020) and a sense of humor (mean 0.82, SD 0.78 vs mean 1.16, SD 0.67, *P*=.003) and more easily accept a stressful situation (mean 1.78, SD 0.77 vs mean 2.11, SD 0.63, *P*=.002), although they tend to experience more negative emotions, which they try to let go of (mean 1.01, SD 0.69 vs mean 1.30, SD 0.67, *P*=.011). This in turn gives the patients with T1DM in our study population a significantly higher level of self-efficacy, as measured with the GSES (mean 27.32, SD 5.32 vs mean 31.08, SD 2.99, *P*<.001).

Worth noticing is the observation that only women from the study population (n=6, 16%) aged 41-54 years had a higher level of state anxiety than people from the general population, as measured with the STAI (mean 47.80, SD 9.78 vs mean 39.50, SD9.27, *P*=.034).

When analyzing the attitude of the patients toward doctors and toward the chronic illness itself, it turned out that the study population accept their illness significantly more than patients with diabetes from the general population, as indicated by AIS scores (mean 24.81, SD 7.09 vs mean 32.17, SD 5.96, *P*<.001). Interestingly, the patients from our study have less internal control over health, that is, they feel that their health does not depend on them as much as expressed by people from the general population. This was indicated in both age groups (26-35 years: mean 24.81, SD 7.09 vs mean 32.17, SD 5.96, *P*<.001; >35 years: mean 24.87, SD 5.71 vs mean 23.57, SD 2.37, *P*=.012). Furthermore, our study participants aged 26-35 years feel significantly more that their health depends on others, especially on their doctors and medical personnel, compared to people from the general population (mean 20.00, SD 4.61 vs mean 24.50, SD 4.07, *P*=.003). In turn, the study participants older than 35 years often consider their health status as the result of chance compared to people from the general population (mean 21.05, SD 6.29 vs mean 17.86, SD 6.18, *P*=.008). In terms of their expectations toward doctors (PRF), the study participants expressed less need to gain information (mean 9.68, SD 2.95 vs mean 8.09, SD 2.86, *P*=.001) and explanations about their health (mean 10.09, SD 2.79 vs mean 8.2, SD 3.27, *P*=.001) that usually people from the general healthy population expect from their doctors.

The overall life satisfaction of patients with T1DM who participated in the study seems to be similar to that declared by people from the general healthy population (mean 20.37, SD 5.32 vs mean 21.46, SD 4.53, *P*=.161). All these comparisons are presented in Table 2.

**Table 2:** Outcomes of psychological tests: comparison of study participants with the general population.

Questionnaire		General healthy population				Studied population of patients with T1DM^a^ at baseline (N=37)			*P* value^b^
		People, n (%)	Mean (SD)		Patients, n (%)		Mean (SD)		
**CISS^c^**									
	Task-oriented score	151 (100)		56.95 (8.65)		34 (92)^d^	58.09 (10.33)		.581
	Emotion-oriented score^a^	151 (100)		45.16 (9.94)		34 (92)^d^	36.94 (10.65)		<.001
	Avoidance-oriented score	151 (100)		43.54 (8.64)		34 (92)^d^	40.78 (9.05)		.105
	Distraction score^a^	151 (100)		19.11 (5.51)		34 (92)^d^	16.34 (4.40)		.002
	Social diversion score	151 (100)		16.42 (3.62)		34 (92)^d^	16.44 (4.09)		.979
**Brief-COPE^e^**									
	Active coping^a^	590 (100)		1.57 (0.79)		37 (100)	2.22 (0.69)		.003
	Planning^a^	590 (100)		1.89 (0.79)		37 (100)	2.30 (0.69)		.005
	Positive reframing^a^	590 (100)		1.67 (0.77)		37 (100)	1.89 (0.54)		.020
	Acceptance^a^	590 (100)		1.78 (0.77)		37 (100)	2.11 (0.63)		.002
	Humor^a^	590 (100)		0.82 (0.78)		37 (100)	1.16 (0.67)		.003
	Religion	590 (100)		0.85 (0.85)		37 (100)	0.87 (0.95)		.900
	Use of emotional support	590 (100)		1.66 (0.91)		37 (100)	1.93 (0.78)		.043
	Use of instrumental support	590 (100)		1.56 (0.93)		37 (100)	1.83 (0.8)		.049
	Self-distraction	590 (100)		1. 37 (0.84)		37 (100)	1.33 (0.61)		.925
	Denial	590 (100)		0.63 (0.71)		37 (100)	0.49 (0.51)		.115
	Venting^a^	590 (100)		1.01 (0.69)		37 (100)	1.30 (0.67)		.011
	Substance use	590 (100)		0.37 (0.65)		37 (100)	0.32 (0.50)		.563
	Behavioral disengagement	590 (100)		0.58 (0.06)		37 (100)	0.57 (0.50)		.907
	Self-blame	590 (100)		1.20 (0.76)		37 (100)	1.04 (0.8)		.237
**STAI^f^**									
	STAI X1 score (males, age 21-40 years)	89 (100)		37.25 (8.65)		13 (35)^g^	38.83 (10.77)		.613
	STAI X2 score (males, age 21-40 years)	89 (100)		39.46 (7.06)		13 (35)^g^	40.83 (9.01)		.599
	STAI X1 score (males, age 41-54 years)	73 (100)		38.16 (8.53)		7 (19)^g^	39.33 (8.08)		.716
	STAI X2 score (males, age 41-54 years)	73 (100)		42.20 (7.62)		7 (19)^g^	37.67 (9.29)		.211
	STAI X1 score (females, age 21-40 years)	90 (100)		36.89 (8.37)		9 (24)^g^	38.00 (8.93)		.699
	STAI X2 score (females, age 21-40 years)	90 (100)		43.27 (8.06)		9 (24)^g^	38.67 (12.19)		.268
	STAI X1 score (females, age 41-54 years)	97 (100)		41.07 (11.05)		6 (16)^g^	36.00 (7.18)		.106
	STAI X2 score^a^ (females, age 41-54 years)	97 (100)		47.80 (9.78)		6 (16)^g^	39.50 (9.27)		.034
GSES^h^ score^a^		496 (100)		27.32 (5.32)		37 (100)	31.08 (2.99)		<.001
PSS-10^i^ score^a^		1830 (100)		16.62 (7.50)		37 (100)	22.14 (4.27)		<.001
SWLS^j^ score		555 (100)		20.37 (5.32)		37 (100)	21.46 (4.53)		.161
AIS^k^ score^a^		70 (100); data for the general population with diabetes		24.81 (7.09)		37 (100)	32.17 (5.96)		<.001
**MHLC ^l^ Scale Form C**									
	Internality score (age 26-35 years)^a^	70 (100)		27.81 (4.48)		8 (22)^m^	24.38 (3.18)		.008
	Doctors and other (powerful) people score (age 26-35 years)^a^	70 (100)		20.00 (4.61)		8 (22)^m^	24.50 (4.07)		.003
	Chance score (age 26-35 years)	70 (100)		16.93 (5.60)		8 (22)^m^	19.0 (6.57)		.392
	Internality score (age>36 years)^a^	485 (100)		24.87 (5.71)		28 (76)^m^	23.57 (2.37)		.012
	Doctors and other (powerful) people score (age>36 years)	485 (100)		23.43 (5.88)		28 (76)^m^	24.14 (4.26)		.403
	Chance score (age>36 years)^a^	485 (100)		21.05 (6.29)		28 (76)^m^	17.86 (6.18)		.008
**PRF^n^**									
	Awaiting clarification of the disease^a^	185 (100)		10.09 (2.79)		37 (100)	8.2 (3.27)		.001
	Searching for emotional support	185 (100)		5.64 (3.97)		37 (100)	4.91 (3.35)		.243
	Gaining information^a^	185 (100)		9.68 (2.95)		37 (100)	8.09 (2.86)		.001

^a^T1DM: type 1 diabetes mellitus.

^b^*P*<.05.

^c^CISS: Coping Inventory for Stressful Situations.

^d^Here, 3 (8%) persons were excluded from the analysis: 1 (33 %) from the 16-24–year and 2 (67%) from the 55-79–year age group. CISS norms for the general population are divided into group categories.

^e^Brief-COPE: Brief Coping Orientation to Problems Experienced Inventory.

^f^STAI: State-Trait Anxiety Inventory.

^g^Here, 1 (3%) female and 1 (3%) male were excluded from the analysis, from the 55-69–year age group. STAI norms for the general population are divided into gender and age categories.

^h^GSES: Generalized Self-Efficacy Scale.

^i^PSS-10: Perceived Stress Scale 10 Items.

^j^SWLS: Satisfaction with Life Scale.

^k^AIS: Acceptance of Illness Scale.

^l^MHLC: Multidimensional Health Locus of Control.

^m^Here, 1 (3%) person was excluded from the analysis, from the 18-25–year age group. MHLC norms for the general population are divided into age categories.

^n^PRF: Patient Requests Form.

### Results of the Second Part of the Analysis

A comparison between the 2 groups created within the study patients with T1DM after randomization and after 3 months from transition (AHCL group with the control group) at the end of the study are presented here.

The AHCL group reported an increase in the QoL in 4 scales: feeling well, which indicates global well-being (score 2.3, 95% CI 0.1-4.6, *P*=.042); working, which is a manifestation of work satisfaction, flexibility at work, and the possibility to fulfill all tasks at work (score 2.8, 95% CI 0.7-4.9, *P*=.012); eating as I would like, which shows emotional freedom in terms of eating, pleasure connected with eating, and the flexibility of eating (score 3.1, 95% CI 0.8-5.4, *P*=.011); and doing normal things, which shows the emotional subjective feeling of a given person, that they may function normally, not being limited by the disease (score 2.8; 95% CI 0.2-5.4, *P*=.034). The level of anxiety significantly decreased in the AHCL group: STAI X1 scores showed that the AHCL group was less anxious in a given moment (score –6.8, 95% CI –11.8 to –1.8, *P*=.009), as confirmed by STAI X1 stens (score –1.4, 95% CI –2.5 to –0.3, *P*=.013). In addition, STAI X2 scores showed that the general, long-term level of anxiety turned out to be lower in the AHCL group (score –3.5, 95% CI –6.5 to –0.5, *P*=.022). Furthermore, the AHCL group became more emotion oriented, which shows they unblocked their emotions when in a stressful situation (CISS E; score 1.1, 95% CI –2.2 to 0.0, *P*=.043) and significantly less tend to self-blame after 3 months of the study (score –0.5, 95% CI –0.9 to –0.09, *P*=.020). All between-groups differences were adjusted for baseline values (Table 3).

**Table 3:** Outcomes of psychological tests: comparison of treatment and control groups at baseline and at the end of the study.

Category		Treatment arm			Control arm			Estimated difference (AHCL^a^ – MDI^b^)^c^		95% CI		*P* value
		Baseline	MiniMed 780G AHCL	Baseline		MDI+SMBG^d^						
**CISS^e^**												
	Task-oriented score	60.67 (7.59)	58.44 (11.24)	53.75 (13.53)		54.75 (13.59)	4.0^f^		–10 to 2.0		.213	
	Task-oriented sten	6.44 (1.76)	6.11 (2.00)	5.33 (2.32)		5.53 (1.88)	1.0^f^		–10.0 to 2.0		.237	
	Emotion-oriented score	37.33 (9.47)	32.78 (7.04)	36.5 (11.51)		37.12 (13.17)	–4.9		–10.4 to 0.6		.077	
	Emotion-oriented sten^g^	4.06 (1.83)	3.17 (1.29	3.88 (2.09)		4.19 (2.59)	–1.1		–2.2 to 0.0		.043	
	Avoidance-oriented score	37.33 (9.47)	39.56 (5.96)	40.88 (10.89)		36.75 (10.02)	2.8		–2.1 to 7.8		.249	
	Avoidance-oriented sten	4.83 (1.92)	4.50 (1.34)	5.13 (2.26)		4.40 (1.84)	0.2		–0.7 to 1.2		.602	
	Distraction score	15.94 (4.17)	15.61 (4.65)	16.94 (4.80)		15.00 (4.65)	0.9		–1.8 to 3.7		.506	
	Distraction sten	4.28 (1.41)	4.28 (1.07)	4.75 (1.84)		4.00 (1.67)	0.4		–0.6 to 1.3		.468	
	Social diversion score	17.00 (3.30)	16.50 (3.70)	15.80 (4.90)		14.80 (4.50)	2.0^f^		–1 to 4.0		.243	
	Social diversion sten	5.70 (1.50)	5.60 (1.60)	5.10 (2.30)		4.80 (2.10)	0.5		–0.7 to 1.6		.408	
**Brief-COPE^h^**												
	Active coping	2.41 (0.48)	2.26 (0.36)	2.00 (0.80)		2.20 (0.62)	–0.1		–0.4 to 0.1		.278	
	Planning	2.44 (0.60)	2.28 (0.45)	2.10 (0.81)		2.00 (0.57)	0^f^		–0.5 to 0.5		.350	
	Positive reframing	2.00 (0.52)	2.28 (0.45)	1.80 (0.62)		1.73 (0.82)	0.5^f^		0 to 1		.094	
	Acceptance	2.28 (0.55)	2.28 (0.48)	2.03 (0.74)		2.00 (0.57)	0.2		–0.2 to 0.5		.316	
	Humor	1.16 (0.81)	1.03 (0.69)	1.10 (0.47)		1.23 (0.56)	–0.2		–0.5 to 0.04		.091	
	Religion	0.97 (1.04)	1.16 (1.08)	0.93 (0.92)		0.93 (0.94)	0.2		–0.2 to 0.6		.287	
	Use of emotional support	2.09 (0.67)	2.06 (0.61)	1.78 (0.93)		1.72 (0.89)	0.2		–0.3 to 0.6		.470	
	Use of instrumental support	2.00 (0.61)	2.00 (0.52)	1.60 (1.04)		1.63 (0.92)	0.8		–0.2 to 1.9		.570	
	Self-distraction	1.38 (0.53)	1.44 (0.48)	1.27 (0.65)		1.10 (0.51)	0.3		–0.04 to 0.6		.079	
	Denial	0.38 (0.45)	0.38 (0.45)	0.62 (0.56)		0.66 (0.60)	–0.2		–0.5 to 0.2		.341	
	Venting	1.34 (0.57)	1.16 (0.54)	1.33 (0.77)		1.33 (0.49)	–0.2		–0.5 to 0.1		.261	
	Substance use	0.32 (0.43)	0.29 (0.44)	0.25 (0.41)		0.22 (0.41)	0^f^		0 to 0.5		.577	
	Behavioral disengagement	0.53 (0.46)	0.69 (0.57)	0.60 (0.57)		0.63 (0.52)	0.1		–0.3 to 0.5		.706	
	Self-blame^g^	1.09 (0.88)	0.81 (0.68)	0.83 (0.67)		1.13 (0.85)	–0.5		–0.9 to –0.09		.020	
**STAI^i^**												
	STAI X1 score^g^	38.00 (9.30)	34.50 (7.80)	35.00 (8.80)		38.70 (12.90)	–6.8		–11.8 to –1.8		.009	
	STAI X1 sten^g^	5.20 (2.20)	4.60 (2.00)	4.50 (2.30)		5.30 (3.00)	–1.4		–2.5 to –0.3		.013	
	STAI X2 score^g^	41.00 (8.50)	37.60 (8.40)	37.90 (9.50)		38.30 (9.70)	–3.5		–6.5 to –0.5		.022	
	STAI X2 sten	5.00 (2.50)	4.30 (2.70)	4.10 (2.30)		4.50 (2.20)	<0.001^f^		–1 to 2.0		.589	
**GSES^j^**												
	GSES score	30.45 (2.07)	31.82 (3.89)	31.75 (3.72)		30.25 (5.74)	2.7		–1.1 to 6.5		.156	
	GSES sten	6.91 (0.83)	7.27 (1.35)	7.17 (1.4)		6.67 (2.27)	0.8		–0.6 to 2.2		.226	
**PSS-10^k^**												
	PSS-10 score	22.58 (3.04)	21.79 (3.22)	21.56 (5.55)		21.75 (4.14)	–0.25		–2.7 to 2.1		.833	
	PSS-10 sten	7.47 (0.90)	7.16 (0.96)	7.12 (1.67)		7.19 (1.42)	–0.1		–0.9 to 0.6		.717	
**SWLS^l^**												
	SWLS score	20.72 (3.29)	21.94 (4.58)	21.56 (4.98)		21.69 (6.11)	1.0		–1.9 to 3.8		.496	
	SWLS sten	5.67 (1.08)	6.11 (1.71)	5.81 (1.83)		6.00 (2.19)	–.2		–0.08 to 1.3		.662	
AIS^m^ score		31.79 (6.01)	32.89 (5.74)	32.12 (5.94)		30.88 (6.11)	11.4		–6.6 to 29.5		.171	
**MHLC^n^ Scale Form C**												
	Internality score	26.16 (4.50)	25.89 (4.88)	24.12 (6.4)		25.09 (5.09)	0.47		–3.0 to 4.0		.785	
	Doctors and other (powerful) people score	25.21 (5.64)	24.16 (4.21)	23.81 (4.74)		23.53 (5.82)	–0.1		–3.0 to 2.8		.928	
	Chance score	18.11 (5.92)	16.58 (5.15)	20.44 (5.98)		21.69 (6.11)	–1.4		–4.0 to 1.2		.283	
**PRF^o^**												
	Awaiting clarification of the disease	9.25 (2.91)	7.88 (3.74)	7.46 (3.67)		9.00 (3.24)	1^f^		01 to 4.0		.369	
	Searching for emotional support	5.67 (3.2)	4.67 (4.06)	5.12 (4.29)		4.88 (3.40)	–0.3		–4.3 to 3.6		.854	
	Gaining information	8.94 (2.43)	8.38 (2.85)	7.27 (3.33)		8.13 (3.36)	–0.8		–2.8 to 1.2		.425	
**QoL-Q Diabetes^p^**												
	Global QoL^q^ score	187.20 (32.00)	202.5 0(54.20)	173.87 (46.24)		173.60 (53.34)	19.9		–17.7 to 57.6		.287	
	QoL family relationships/friendships	9.90 (3.40)	10.00 (3.50)	8.27 (3.13)		10.20 (3.49)	<–0.001^f^		–2.99 to 2.0		.604	
	QoL going out or socializing	8.20 (2.70)	8.00 (2.80)	8.07 (3.43)		9.33 (2.97)	–1.02		–3.1 to 1.0		.311	
	QoL partner/spouse relationship	9.80 (3.00)	9.70 (4.00)	9.53 (3.34)		9.73 (4.10)	–0.1		–3.0 to 2.8		.943	
	QoL enjoying sexual activity	8.40 (2.30)	8.80 (3.10)	8.71 (2.43)		9.21 (3.91)	–0.32		–2.9 to 2.3		.797	
	QoL being physically active	80 (2.10)	8.80 (2.80)	7.47 (3.14)		8.36 (3.08)	0.71		–1.7 to 3.1		.544	
	QoL feeling well^g^	7.90 (3.20)	9.80 (3.10)	6.73 (3.31)		7.87 (3.38)	2.3		0.1 to 4.6		.042	
	QoL feeling in control of my body	7.90 (2.50)	8.70 (3.40)	7.50 (2.44)		8.93 (3.59)	<–0.001^f^		–2.0 to 2.0		.801	
	QoL looking good	7.20 (2.20)	7.70 (2.50)	7.79 (2.78)		8.20 (3.38)	0.3		–1.6 to 2.3		.718	
	QoL having holidays	8.90 (3.50)	9.30 (3.40)	6.93 (3.01)		7.53 (2.59)	1.3		–0.9 to 3.5		.234	
	QoL working^g^	7.40 (2.00)	10.40 (2.90)	7.93 (4.15)		7.33 (3.02)	2.7		0.7 to 4.6		.010	
	QoL affording the things I would like	8.70 (2.70)	8.90 (3.60)	8.20 (3.47)		8.07 (3.20)	1.4		–0.9 to 3.7		.214	
	QoL driving	8.60 (3.40)	9.10 (3.20)	7.80 (4.18)		7.93 (3.79)	0.9		–1.2 to 3.1		.393	
	QoL practicing my religion	8.40 (3.90)	8.40 (3.80)	7.18 (4.33)		6.30 (3.92)	0.9		–0.7 to 2.6		.249	
	QoL sleeping	8.60 (2.50)	9.8 (3.3)	7.27 (2.09)		7.50 (4.31)	2.1		–0.3 to 4.5		.087	
	QoL eating as I would like^g^	4.90 (2.70)	7.1 (3.3)	3.87 (1.88)		4.07 (2.62)	3.1		0.8 to 5.4		.011	
	QoL looking after or being useful to others	8.90 (2.20)	8.7 (1.9	7.33 (2.47)		7.31 (3.30)	2.0^f^		–4.5 to 4.0		.081	
	QoL pets/animals	7.70 (1.90)	8.80 (3.70)	7.09 (2.88)		6.44 (2.35)	2.1		–0.1 to 4.4		.065	
	QoL being independent	9.70 (3.20)	11.10 (3.10)	9.80 (2.93)		9.62 (3.73)	1.5		–0.7 to 3.8		.178	
	QoL being in control of my life	9.20 (3.20)	10.60 (3.50)	9.20 (3.21)		8.57 (4.26)	1.7		–1.0 to 4.5		.208	
	QoL being spontaneous	6.50 (2.70)	6.50 (2.70)	6.67 (3.62)		6.67 (3.62)	1^f^		–2.0 to 4.0		.430	
	QoL doing a “normal” thing^g^	7.60 (2.90)	9.90 (3.70)	8.33 (3.66)		8.21 (3.85)	2.8		0.2 to 5.4		.034	
	QoL being treated as “normal”	9.00 (2.90)	9.30 (4.10)	9.40 (3.14)		9.36 (4.29)	0.5		–2.7 to 3.7		.750	
	QoL having confidence	9.00 (2.20)	9.30 (3.30)	8.20 (3.21)		8.29 (4.10)	–0.9		–2.8 to 0.9		.310	

^a^AHCL: Advanced Hybrid Closed Loop.

^b^MDI: multiple daily injection.

^c^Analysis of covariance (ANCOVA) with adjustment for the baseline value. The mean difference is presented.

^d^SMBG: self-monitoring of blood glucose.

^e^CISS: Coping Inventory for Stressful Situations.

^f^Wilcoxon rank-sum test was applied when ANCOVA assumptions were not meet.

^g^*P*<.05.

^h^Brief-COPE: Brief Coping Orientation to Problems Experienced Inventory.

^i^STAI: State-Trait Anxiety Inventory.

^j^GSES: Generalized Self-Efficacy Scale.

^k^PSS-10: Perceived Stress Scale 10 Items.

^l^SWLS: Satisfaction with Life Scale.

^m^AIS: Acceptance of Illness Scale.

^n^MHLC: Multidimensional Health Locus of Control.

^o^PRF: Patient Requests Form.

^p^QoL-Q Diabetes: Quality of Life in Diabetes Questionnaire.

^q^QoL: quality of life.

## Discussion

### Principal Findings

Our study aimed at evaluating adult patients with T1DM who after many years of MDI treatment and SMBG using a blood glucose meter (BGM) had their treatment changed directly to the MiniMed 780G AHCL system. First, we showed that in comparison to the general healthy population, our studied population before the transition was characterized by a higher level of stress but at the same time higher self-efficacy and better coping strategies. Furthermore, we revealed that during the first 3 months of the treatment change from MDI and SMBG directly to the AHCL system, the patients experienced improvement in various aspects of QoL and also experienced a decrease in anxiety and the feeling of guilt. In addition, the mental well-being of the transitioned patients did not deteriorate in any of the examined patients. In the control group, the examined parameters stayed unchanged.

We decided to carry out a 2-stage analysis: first to check the general psychological parameters of all the patients with T1DM who enrolled in the study. This was an interesting population of patients who had never before decided to use modern technologies in their diabetes management and now they had decided to overcome their earlier habits and attitudes toward modern technologies. We wanted to make a comparison of their psychological characteristics to the general Polish population and then check the between-group differences after the randomization process and 3-month study, when one part of the group underwent the transition in treatment and the other part became the control group, without a change in treatment. From a psychological point of view, this is a significant change in terms of diabetes control—the level of responsibility of glucose level control, calculation of the insulin dosage, and reactions to the blood glucose level resulting from a proper medical decision 24 hours a day shifted to the insulin pump, which is highly automatized. Thanks to the automation, the pump reacts independently of the patient to glucose level changes, protecting the patient from both hypo- and hyperglycemia. This requires a change in the control level that the patients got used to over many years of treatment [10,11]. To the best of our knowledge, there is no study carried out so far that has examined in a direct way the psychological reaction to a direct change of treatment from the traditional way of treatment to the most modern one available.

Another change concerns the self-image. Treatment with insulin injections with the use of a pen and monitoring the glucose blood level with a traditional BGM usually require several measurements and a few injections per day. The MiniMed 780G AHCL system is connected with a permanent personal insulin pump, sensor, transmitter, CGMS, and infusion set being placed on the body. The system has alarms set, which can be heard by people around. We assumed that for some patients, this kind of change might not have any special meaning and they would get used to it easily, but for others, it could be connected with the need to overcome some level of resistance, discomfort, or even shame resulting from—for some of the patients—the specific coming out of being diabetic, for example, in the workplace [27,28,30-33]. Nowadays, children and adolescents are usually treated with modern technologies (personal insulin pump, CGMS) immediately after being diagnosed or soon after the diagnosis. The same is offered to adults diagnosed with T1DM, although in this group of patients, finances are often a limitation. The patients in our study were treated with traditional intensive insulin therapy for many years before—some of them for over 30 years. They developed their identity around the traditional method of treatment, and the possibility to use the MiniMed 780G insulin pump was a real-life breakthrough [30,31].

Analyzing the initial psychological parameters of the patients who enrolled in the study, we observed that this group of patients had good coping strategies to deal with stress—to some extent even more efficient than the general population. We believe that the patients worked out stress adjustment during many years of dealing with diabetes, and at the same time, perhaps those psychological resources encouraged them to apply for participation in the study and then allowed optimal adjustment to the new method of treatment. Our results also indicate that in terms of diabetes education, the patients expressed no special needs apart from learning how to use the new pump/CGMS.

We applied as many as 10 questionnaires to make sure that we properly monitored various aspects of the patients’ psychological functioning. None of the patients from the study reacted negatively, with aroused levels of stress or inconvenience. The level of stress, ways of dealing with difficult situations, and acceptance of the illness did not have any negative response in comparison to the control group after 3 months of treatment. In addition, after the 3-month period of application of the MiniMed 780G AHCL system, the level of anxiety—both state anxiety and trait anxiety—was significantly lower than in the control group. This is especially important when we consider the fact that for many patients with T1DM, the source of constant anxiety is the possibility of having hypoglycemia during various daily activities, as indicated by researchers [32-35], but also, they often face fear connected with the possibility of developing late complications [36]. Thus, the decrease in anxiety may result not only from the protection from hypoglycemia, which is ensured by the closed-loop system, but also from the awareness that the TIR, which was significantly higher in this group, allows the patients to get more confidence in their long-term better health and lower risk of late complications.

Essential is the significant increase in the QoL of the AHCL group in 4 important areas: feeling well, working, eating as I would like, and doing normal things.

Knowledge about the QoL is important for understanding the consequences of illness and treatment and for medical decision-making across age groups and cultures. The QoL is an important end point in medical and health research, and QoL research involves a variety of target groups and research designs [37]. Numerous demographic and psychosocial factors influence the QoL. Studies of clinical and educational interventions suggest that improving patients' health status and perceived ability to control their disease results in improved QoL [38-40].

Our study revealed that not only did a change in the treatment method from MDI and SMBG with a BGM directly to the MiniMed 780G AHCL system not worsen the subjective assessment of the QoL of the patients, but they also felt better in a significant way in general, experienced improvement in their working place, felt more “normal and free” in their everyday eating, and could more easily state that they can do normal things. In addition, the patients from the AHCL group experienced less feelings of guilt at the end of the study and could more easily be in contact with their emotions in stressful situations. This may relate to the fact that the improvement in diabetes control and shift in responsibility for glucose level results from the patient to the highly automatized insulin pump unblocked the burden of responsibility the patients had to deal with before and allowed them to be more focused on emotions connected with a given life situation and not so much on diabetes.

All the metabolic parameters and changes obtained during the study in terms of glucose control (TIR, HbA_1c_, etc) are presented in Ref. [7].

### Limitations of the Study

One limitation of the study was the short period of observation (3 months), but as the study continues, we will keep on monitoring the psychological parameters and conduct further analyses after a year of study progress. In addition, when drawing conclusions connected with possible psychological outcomes connected to transition from MDI and SMBG treatment directly to the MiniMed780G AHCL system in patients with T1DM naive to technology, we must consider the fact that the patients in our studied group were highly motivated and had good psychological resources. Furthermore, they had regular control visits and could easily consult with the medical team about all their doubts (the frequency of visits was higher in comparison to standard therapy). In real-life conditions, this is not always easily available.

Another limiting factor was the number of comparisons made, and it is likely that type 1 errors may have occurred. Considering psychological parameter change/analysis, the studied group was relatively small. In addition, questionnaires were not administered in random order, so patient fatigue may have influenced the results.

### Conclusion

The results indicate that the patients who decided to take part in the transition study were characterized by a higher level of stress than the general healthy population but had better coping strategies and self-efficacy. Furthermore, we can conclude that transition from MDI and SMBG treatment directly to the MiniMed 780G AHCL system in patients with T1DM naive to technology can not only in a significant way improve glucose control and metabolic parameters fast as after 3 months but also in a noticeable way improve the well-being of patients, giving them more comfort, freedom, and life flexibility. The rapidity of these changes suggests that they may be related to significant improvement in glycemic outcomes but also significantly less burdened diabetes self-management.

## Abbreviations

AHCL: advanced hybrid closed loop
AIS: Acceptance of Illness Scale
ANCOVA: analysis of covariance
BGM: blood glucose meter
Brief-COPE: Brief Coping Orientation to Problems Experienced Inventory
CGMS: continuous glucose-monitoring system
CISS: Coping Inventory for Stressful Situations
CSII: continuous subcutaneous insulin infusion
GSES: Generalized Self-Efficacy Scale
HbA_1c_: hemoglobin A_1c_
HCL: hybrid closed loop
MDI: multiple daily injection
MHLC: Multidimensional Health Locus of Control
PRF: Patient Requests Form
PSS-10: Perceived Stress Scale 10 Items
QoL: quality of life
QoL-Q Diabetes: Quality of Life in Diabetes Questionnaire
SMBG: self-monitoring of blood glucose
STAI: State-Trait Anxiety Inventory
SWLS: Satisfaction with Life Scale
T1DM: type 1 diabetes mellitus
TBR: time below range
TIR: time in range

## Data Availability

Data are available upon reasonable request from the corresponding author.
